# Childhood Cancer Survival, 2006-2012 Cohorts of Mexican Institute of Social Security Beneficiaries at the Central-South Region of Mexico

**DOI:** 10.3389/fonc.2022.882501

**Published:** 2022-07-01

**Authors:** Angélica Castro-Ríos, Silvia Martínez-Valverde

**Affiliations:** ^1^ Unidad de Investigación en Epidemiología Clínica, Hospital de Pediatría, Centro Médico Nacional Siglo XXI, Instituto Mexicano del Seguro Social, Ciudad de México, Mexico; ^2^ Centro de Estudios Económicos y Sociales en Salud, Hospital Infantil de México Federico Gómez, Instituto Nacional de Salud, Ciudad de México, Mexico

**Keywords:** childhood cancer, survival, Mexico, social security, cohorts, IMSS

## Abstract

**Introduction:**

In Mexico, the main institution of social security is the “Instituto Mexicano del Seguro Social” (IMSS), with more than 60 million enrolled individuals. This study of childhood cancer survival is the first based on complete cohorts of incident cases for the population IMSS- affiliated in the central-south region, which represents 27% of all children IMSS affiliated.

**Methods:**

It is an observational cohort study from 2006 to 2012 to estimate the 5-year observed survival of the minors under 18 years old, identified in the Central-South Region Registry of Children with Cancer. The survival of cases was carried out through the active and passive search. Survival rates were estimated by the Kaplan–Meier (KM) method, the analysis of equality of survival functions was evaluated for some clinical variables.

**Results:**

The study included 2,357 minors; the 5-year observed survival was 56.1% with a time of survival median of 3.4 years, and the overall loss of follow-up was 18.4%. The 5-year survival in cases with a diagnosis of leukemia was 53.5%, while for solid tumors, it was 57.9%. The median time of death was 1 year. The types of cancer with a survival greater than 70% were group V-retinoblastoma (87.2%), IIa-Hodgkin’s lymphoma (86.8%), Xc- gonadal tumors (83.3%), Iid-miscellaneous lymphomas (80%), IVa-nephroblastoma (79.5%), and IIc-Burkitt’s lymphoma (75.4%). Meanwhile, the lowest survival rates were in group VIII-bone tumors (32.3%), III-CNS (central nervous system; 44.1%), and IX-soft tissues (46.8%).

**Conclusions:**

Survival results in the 2006–2012 cohorts show a significant gap in relation to the goal of 60% proposed by the World Health Organization for 2030.

## Introduction

It is estimated that each year, approximately 430,000 individuals under 20 years of age will develop some type of cancer with 10.5% of cases in high-income countries and 89.5% in low- and middle- income countries (1). Technological advances have allowed changes in treatment strategies and improvement in survival (2), and, although the cure rate across high-income countries exceeds 80%, across low- and middle-income countries, it is below 50% (3). The causes of poor survival have been attributed to delays in diagnosis and advanced disease, as well as in the inability to obtain an accurate diagnosis, inaccessible therapy, the abandonment of treatment, death from toxicity, and avoidable relapse (3).

In September of 2018, the World Health Organization (WHO) launched the Global Initiative for Childhood Cancer, with the goal that by the year 2030, at least 60% survival would be achieved in those under 20 years of age (4).

In high-income countries, information on cancer incidence and survival is routinely collected and analyzed. Large regional studies such as EUROCARE (5) or those of the United States of America coordinated by the Surveillance, Epidemiology, and End Results (SEER) (6, 7), have provided information for decades. Meanwhile, in low- and middle-income countries, information is scarce (8, 9).

In Mexico, in recent years, cancer registry initiatives have been implemented, focusing on the incidence registry. One of the oldest is the Registry of children with cancer of the central region (RCC) of the Mexican Institute of Social Security [Instituto Mexicano del Seguro Social (IMSS)], which began in 1996 (10). In 2017, for the first time, data on the incidence of children with cancer in Mexico were included in the International Agency for Research on Cancer (IARC) report “International Incidence of Childhood Cancer” (11) because the data met the quality standards required for this purpose (12).

On the other hand, the childhood cancer survival data available in Mexico is derived from research about specific conditions or treatments (13) and some sporadic reports (14). With regard to that, in 2015, the RCC of the IMSS started the recollection of data on survival obtained from active and passive search.

Therefore, the objective of this study was to show the results of the 5-year survival rates for cohorts of children diagnosed during the period 2006–2012 registered in the *RCC of the IMSS*.

### Study Context

In Mexico, the main institution of social security is the IMSS with more than 60 million enrolled individuals (15). Individuals are enrolled in the IMSS in either of the two plans: the voluntary or mandatory plan (for formal workers and their families and students in high school or university) (16).

The IMSS has an infrastructure all over the country consisting of approximately 1,500 primary-care units, 270 secondary hospitals, and 30 tertiary hospitals, which are organized into ten medical-service networks (17). The RCC includes two medical networks: the “La Raza” network (8 million people approximately, which includes the population of northern Mexico City and the states of Mexico and Hidalgo) and the SXXI network (6 million people approximately, which includes the population of southern Mexico City and the states of Chiapas, Guerrero, Morelos, and Querétaro), where altogether, almost 14 million people are served and which represents 27% of all children affiliated to the IMSS (15).

## Material and Methods

This was an observational study of cohorts of incident cases from 2006 to 2012, to estimate 5-year observed survival (18). The study population comprised children identified in the RCC.

The RCC of the IMSS collects data for the incident cases of children diagnosed with cancer and generates a file for each of them, which includes information about: clinical staging and the initiation of treatment and an initial interview to the parents for collecting socioeconomic data, pathological antecedents of the child and family, and diagnostic history (including the temporality of signs, symptoms, and care trajectory prior to diagnosis) (19). The variables include in this report are as follows: cancer diagnosis codified according the ICCC-3, age group at cancer diagnosis, sex, the year of diagnosis, and time lag in diagnosis, which refers to the parents’ report of the time elapsed between the appearance of relevant symptoms and the date of diagnosis by the specialist doctor (20).

The registry includes the beneficiaries of the IMSS under 18 years of age with a confirmed diagnosis of cancer that was diagnosed or received partial or total treatment in the third-level hospitals of the two service networks of the region (“La Raza” and the SXXI). It means that only those cases that present in the third-level medical unit with a probable diagnosis of cancer but do not confirm or do not complete the diagnostic process in the unit are excluded from the register of incident cases (19).

The registry includes all children with neoplasms with the International Classification of Diseases for Oncology (ICDO-3) (21) behavior codes/2 (*in situ*) or/3 (malignant primary site) and benign codes/0 and/1 for borderline intracranial and central nervous (CNS) system tumors, following the standards required for cancer registries (12).

The survival time postdiagnosis was calculated in natural days, considering the time elapsed between the cancer diagnosis date and the date of the last contact. For the patients who died within 5 years postdiagnosis, the last contact date corresponds to the date of death; for the cases where follow-up was lost, the date of the last contact was defined as the most recent date between registers in the clinical record, institutional data sources, or the last date the patient was reported alive by the contact family member; for those who survived, the date was truncated at 5 years after diagnosis.

The follow-up of patients included the following: a review of the clinical file of the patients (physical and electronic) at the third-level hospital included in the study; the identification of patients in local data sources (Headquarters of Oncology Services); the identification of patients in national institutional data sources; and reports from a family member registered as responsible (through a home or work phone). The closing date of this study was December 31, 2017.

The identification of cases in national institutional data sources was carried out with the collaboration of the Health Information Directorate and the Hospital Discharge Information System (SUI) and the Mortality System (SISMOR). Due to the fact that patients frequently have multiple social security numbers because to changes in the insurance modality, 3 search algorithms will be considered for the confirmation of the coincidence of the case. The variables including in the search algorithms were as follows: the social security number, full name, year of birth, and International Classification of Diseases (ICD-10) codes of cancer diagnosis.

Death was determined when the patient was identified within dates before 5 years since diagnosis as a) a coincident case based on hospital discharge records due to death, b) a coincident case based on the mortality database, or c) the report for a family member at a medical hospital or by telephone call. Survival was determined when the patient was identified in any date subsequent at 5 years after diagnosis as a) a coincident case discharge based on hospital discharge or b) if the case is reported alive by a family member by telephone call. The status “loss of follow-up” was determined according to these 3 conditions: a) no coincident case based on hospital discharge records any date after 5 years after diagnosis, b) no coincident case based on the mortality database at any date before at 5 years after diagnosis, and c) was not possible to communicate with a family member by telephone call in a date subsequent to 5 years since diagnosis.

### Survival Estimations

The survival time and overall survival (OS) at 5 years were calculated for each patient using the cohort method. To estimate the probability of survival, the tables of life expectancy and survival curves were generated using the Kaplan-Meier (KM) method according to the following formula (18):


$$S\;\left(t\right)\;=\;\mathrm{Pr}\left(T\geq t\right)\text{ > }=\;\prod \left(1\;-\;\frac{d_{i}}{n_{i}}\right)\;,\;with\;T:life\;span\;of\;the\;patient\;diagnosis$$

where di: deaths and ni: alive patients.

The KM method (22) is a statistical treatment for the calculation of survival time, which considers time in many small intervals and therefore uses the information of all the observations up to the moment they are censored, not only the observations with complete follow-ups.

The exploratory analysis of the equality of functions in subpopulations was carried out and was evaluated using the Wilcoxon test (18) for each of the characteristics listed in **Table 1**.

**Table 1:** Variables studied.

Variable	Category
Cancer diagnosis codified according the International Classification of Childhood Cancer, 3rd edition (ICCC-3) (21)	Categorical variable: I. Leukemias, myeloproliferative, and myelodysplastic diseases, II. Lymphomas and reticuloendothelial neoplasms, III. CNS and miscellaneous intracranial and intraspinal neoplasms, IV. Neuroblastoma and other peripheral nervous cell tumors, V. Retinoblastoma, VI. Renal tumors, VII. Hepatic tumors, VIII. Malignant bone tumors, IX. Soft tissue and other extraosseous sarcomas, X. Germ cell and trophoblastic tumors and neoplasms of gonads, XI. Other malignant epithelial neoplasms and malignant melanomas, XII. Other and unspecified malignant neoplasms.
Age group at cancer diagnosis	Years between a minor’s date of birth and date of diagnosis. Ordinal variable: 1) <1 year old; 2) 1 to <5 years old; 3) 5 to <10 years old; 4) 10 to <15 years old, and 5) 15 to <19 years old.
Sex	Dichotomous variable: 1) male; 0) female.
Time lag in diagnosis	Months between parents’ awareness of symptoms and date of diagnosis in a tertiary hospital (20). Ordinal variable: 1) <1 month; 2) [1, 3) months; 3) [3, 6) months, 4) ≥6 months.

Source of information: Register of Childhood Cancers, maintained by Clinical Epidemiology Research Unit-Pediatrics Hospital, contains clinical and socioeconomic data and contact information for minors and their families CNS, central nervous system.

For the comparative purposes, the standardization of rates was carried out for ages [0,15) years and [0, 18) years, applying population data from the WHO (23, 24).

### Ethical Approval and Informed Consent

We obtained the informed consent from the family members responsible for each child, which was requested at the time of registration and at the initial interview. In this talk, it is explained that in the case of giving their consent, the follow-up will be maintained until the treating doctor declares the child discharged, which includes the periods of treatment and surveillance after diagnosis, and, of course, it is explained to them that they can withdraw their consent at any time. Once the diagnosis is confirmed, an appointment is made with a family member to apply a survey to collect a detailed clinical and sociodemographic history of the minor.

Approval for performing the study was obtained from the corresponding Research and Bioethics Committee of the IMSS (registration number 2003/718/070) and registered at the RCC. Likewise, each member of the registry team that worked on this survival study signed a data protection and confidentiality commitment in accordance with the specific recommendations of the IARC cancer registries (12).

## Results

The study included 2,357 children diagnosed during the period 2006–2012. Of these, 43.4% died within 5 years of diagnosis; 87% of the deaths occurred in IMSS facilities and were registered in the institutional mortality system (SISMOR).

The overall loss of follow-up was 18.4%. Statistically significant differences were observed in the rate of loss to follow-up between the group of diagnosis and age categories. The highest rate of loss to follow-up was in the group of diagnosis XI-other epithelial neoplasms (37.1%) and the lowest rate was in the leukemia group (12.4%). A growing pattern was observed between age and the rate of loss to follow-up where the loss rate was greater than 25% in patients over 10 years old.

**Table 2** shows the 5-year KM survival rates, for both the [0–15)-year-old and [0–18)-year-old groups. In general, the 5-year observed survival rate for minors under 18 years old was 56.1% with (53.9%, 58.1%) 95% confidence interval (95% CI), with a median survival time of 3.4 years; for patients with leukemia, the survival rate was 53.5% with (50.2%, 56.7%) 95% CI, while for cases with a solid tumor, survival was 57.9% with (55.1%, 60.6%) 95% CI.

**Table 2:** Five-year survival rate for childhood cancer in Mexican population 2006–2012.

Cancer diagnosis (ICC-3)		Kaplan–Meier Survival rates^a^								Survival	
		[0, 15) years old				[0, 18) years old				years^b^	
		Cases	Rate	[IC 95%]		Cases	Rate	[IC 95%]		All	Died
**All cancers**		**2,184**	**56.6%**	**54.4%**	**58.8%**	**2,357**	**56.1%**	**53.9%**	**58.1%**	**3.4**	**1.0**
**I.**	**Leukemias and myeloproliferative and myelodysplastic diseases**	**885**	**54.5%**	**51.1%**	**57.8%**	**952**	**53.5%**	**50.2%**	**56.7%**	**3.8**	**1.2**
a.	Lymphoid leukemias	735	56.1%	52.3%	59.7%	790	55.0%	51.4%	58.5%	4.2	1.4
b.	Acute myeloid leukemias	132	44.3%	35.3%	52.8%	144	43.8%	35.3%	52.0%	1.4	0.5
c.	Chronic myeloproliferative diseases	4	100.0%	.	.	4	100.0%	.	.	5.0	–
d.	Myelodysplastic syndrome and other myeloproliferative diseases	5	60.0%	12.6%	88.2%	5	60.0%	12.6%	88.2%	1.0	0.8
e.	Unspecified and other specified leukemias	9	52.9%	17.9%	79.1%	9	52.9%	17.9%	79.1%	2.6	0.3
	**Solid tumors**	**1,299**	**58.1%**	**55.2%**	**60.9%**	**1,405**	**57.9%**	**55.1%**	**60.6%**	**3.1**	**1.0**
**II.**	**Lymphomas and reticuloendothelial neoplasms**	**243**	**70.4%**	**63.9%**	**76.0%**	**265**	**70.0%**	**63.8%**	**75.4%**	**5.0**	**1.2**
a.	Hodgkin disease	67	88.1%	76.7%	94.2%	79	86.8%	76.1%	92.9%	5.0	2.1
b.	Non-Hodgkin lymphomas (except Burkitt)	98	52.2%	41.5%	61.9%	105	51.8%	41.4%	61.3%	2.6	1.1
c.	Burkitt lymphoma	29	76.5%	55.0%	88.7%	32	75.4%	55.3%	87.5%	5.0	0.7
d.	Miscellaneous lymphoreticular neoplasms	49	80.0%	65.1%	89.1%	49	80.0%	65.1%	89.1%	5.0	0.4
**III.**	**CNS and miscellaneous intracranial and intraspinal neoplasms**	**316**	**42.7%**	**36.9%**	**48.3%**	**333**	**44.1%**	**38.4%**	**49.6%**	**1.6**	**0.8**
a.	Ependymomas and choroid plexus tumors	47	59.0%	42.6%	72.2%	48	59.5%	43.2%	72.6%	3.1	0.8
b.	Astrocytomas	118	43.4%	33.9%	52.6%	128	45.1%	35.8%	54.0%	1.4	0.9
c.	Intracranial and intraspinal embryonal tumors	99	34.4%	25.0%	43.9%	101	35.4%	26.0%	44.9%	1.5	0.9
d.	Other gliomas	20	22.2%	6.9%	42.9%	21	24.3%	8.2%	44.9%	0.8	0.8
e.	Other specified intracranial and intraspinal neoplasms	24	64.4%	41.3%	80.4%	27	66.7%	44.3%	81.7%	4.8	2.0
f.	Unspecified intracranial and intraspinal neoplasms	8	28.6%	4.1%	61.2%	8	28.6%	4.1%	61.2%	0.7	0.6
**IV.**	**Neuroblastoma and other peripheral nervous cell tumors**	**46**	**51.8%**	**35.8%**	**65.6%**	**46**	**51.8%**	**35.8%**	**65.6%**	**2.9**	**1.1**
a.	Neuroblastoma and ganglioneuroblastoma	45	50.6%	34.5%	64.7%	45	50.6%	34.5%	64.7%	2.7	1.1
b.	Other peripheral nervous cell tumors	1	100.0%	.	.	1	100.0%	.	.	5.0	
**V**	**Retinoblastoma**	**84**	**87.2%**	**77.5%**	**92.9%**	**84**	**87.2%**	**77.5%**	**92.9%**	**5.0**	**0.8**
**VI.**	**Renal tumors **	**104**	**79.0%**	**69.3%**	**85.9%**	**105**	**78.1%**	**68.5%**	**85.2%**	**5.0**	**1.0**
a.	Nephroblastoma and other nonepithelial renal tumors	101	79.5%	69.7%	86.4%	101	79.5%	69.7%	86.4%	5.0	1.0
b.	Renal carcinomas	3	60.0%	2.5%	93.2%	4	42.9%	2.9%	81.6%	3.7	2.6
**VII.**	**Hepatic tumors**	**47**	**56.1%**	**39.7%**	**69.6%**	**47**	**56.1%**	**39.7%**	**69.6%**	**1.7**	**0.8**
a.	Hepatoblastoma	39	55.6%	38.1%	69.9%	39	55.6%	38.1%	69.9%	3.3	0.8
b.	Hepatic carcinomas	7	55.6%	9.1%	86.6%	7	55.6%	9.1%	86.6%	1.0	1.2
c.	Unspecified malignant hepatic tumors	1	100.0%	.	.	1	100.0%				
**VIII.**	**Malignant bone tumors**	**130**	**31.9%**	**23.6%**	**40.5%**	**153**	**32.3%**	**24.6%**	**40.3%**	**1.5**	**1.2**
a.	Osteosarcomas	94	31.3%	21.6%	41.4%	114	32.3%	23.3%	41.6%	1.6	1.3
b.	Chondrosarcomas	1	0.0%	.	.	1	0.0%			0.7	0.7
c.	Ewing tumors and related sarcomas of bone	30	35.7%	18.9%	53.0%	31	34.5%	18.2%	51.5%	1.5	1.0
d.	Other specified malignant bone tumors	5	25.0%	0.9%	66.5%	6	33.3%	2.7%	71.6%	1.4	1.5
e.	Unspecified malignant bone tumors	0				1	0.0%			1.2	1.2
**IX.**	**Soft tissue and other extraosseous sarcomas**	**134**	**46.6%**	**37.4%**	**55.3%**	**143**	**46.8%**	**37.9%**	**55.2%**	**2.0**	**1.1**
a.	Rhabdomyosarcomas	61	50.0%	36.1%	62.4%	62	49.1%	35.4%	61.4%	2.7	1.4
b.	Fibrosarcomas, peripheral nerve sheath, and other fibrous neoplasms	16	57.1%	28.4%	78.0%	17	53.3%	26.3%	74.4%	2.3	1.4
d.	Other specified soft tissue sarcomas	55	41.7%	27.7%	55.0%	62	44.4%	31.0%	57.0%	1.8	0.9
e.	Unspecified soft tissue sarcomas	2	0.0%	.	.	2	0.0%			0.9	1.0
**X.**	**Germ cell tumors, trophoblastic tumors, and neoplasms of gonads**	**155**	**72.6%**	**64.2%**	**79.3%**	**189**	**70.0%**	**62.4%**	**76.4%**	**4.5**	**0.7**
a.	Intracranial and intraspinal germ cell tumors	38	56.5%	38.6%	71.1%	45	51.2%	35.2%	65.2%	2.1	0.6
b.	Malignant extracranial and extragonadal germ cell tumors	13	36.0%	12.2%	60.9%	20	42.1%	20.4%	62.5%	1.5	1.0
c.	Malignant gonadal germ cell tumors	103	83.9%	74.4%	90.1%	122	83.3%	74.5%	89.2%	5.0	1.0
d.	Gonadal carcinomas	0				1	0.0%			0.0	0.0
e.	Other and unspecified malignant gonadal tumors	1	100.0%	.	.	1	100.0%			5.0	–
**XI.**	**Other malignant epithelial neoplasms and malignant melanomas**	**33**	**69.8%**	**48.6%**	**83.6%**	**35**	**64.9%**	**44.6%**	**79.3%**	**3.6**	**1.1**
a.	Adrenocortical carcinomas	3	20.0%	0.1%	70.8%	3	20.0%	0.1%	70.8%	1.4	0.7
b.	Thyroid carcinomas	13	100.0%	.	.	13	100.0%	.	.	5.0	–
c.	Nasopharyngeal carcinomas	2	50.0%	0.6%	91.0%	2	50.0%	0.6%	91.0%	3.1	1.3
d.	Malignant melanomas	0				1	0.0%	.	.	0.6	0.6
f.	Other and unspecified carcinomas	15	54.6%	22.9%	78.0%	16	50.0%	20.9%	73.6%	1.8	1.2
**XII.**	**Other and unspecified malignant neoplasms**	**5**	**55.6%**	**9.1%**	**86.6%**	**5**	**55.6%**	**9.1%**	**86.6%**	**4.1**	**0.8**
a.	Other specified malignant tumors	5	55.6%	9.1%	86.6%	5	55.6%	9.1%	86.6%	4.1	0.8

ICCC-3, International Classification of Childhood Cancer, 3rd edition; CNS, central nervous system. a: Kaplan–Meier estimates with actuarial adjustment, b: Median time of survival since diagnosis and up to 5 years or death. Bold values means to differentiate diagnoses groups from specific diagnosis.

**Figure 1:**
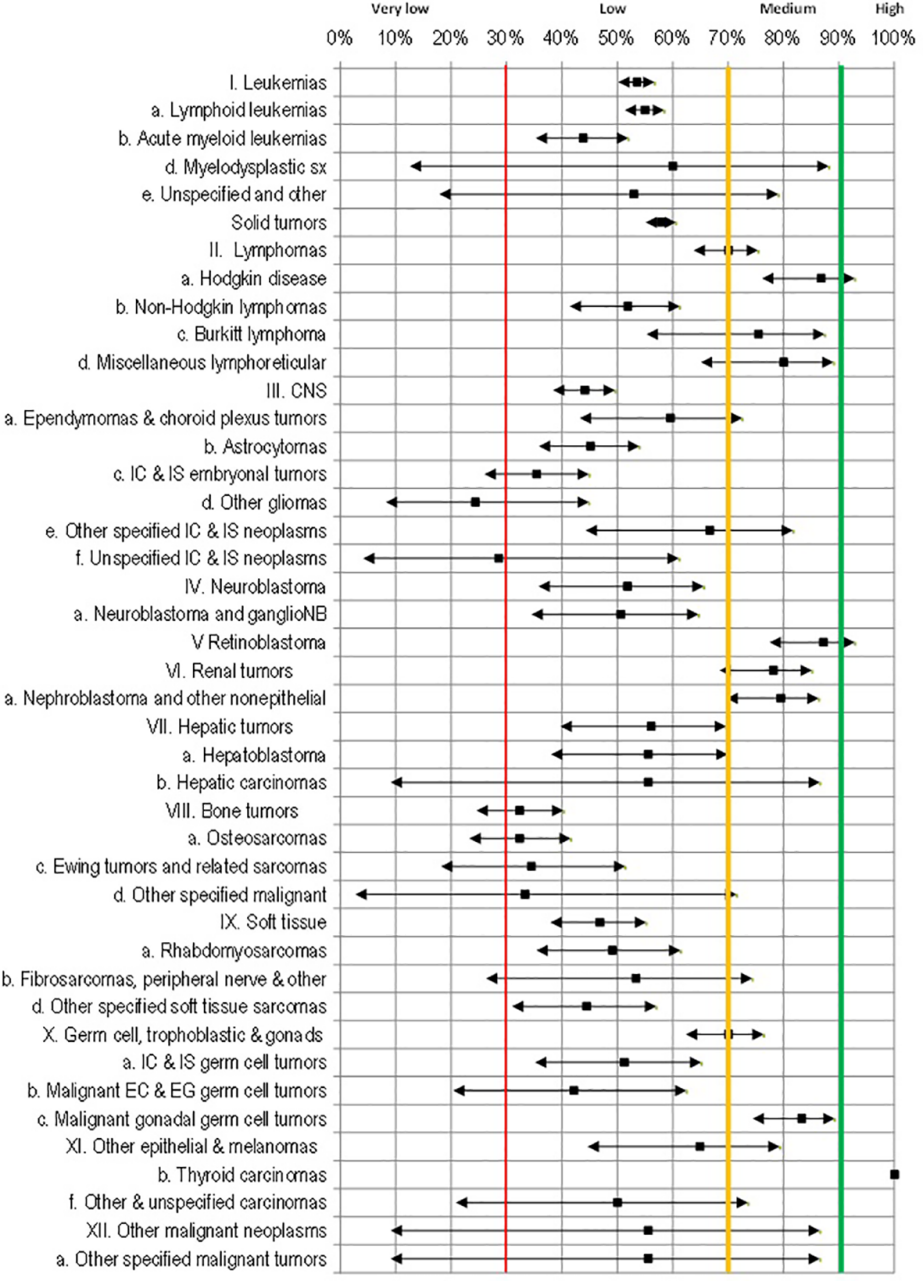
95% confidence intervals for 5-year survival rates by cancer diagnosis.

**Figure 1** shows the survival levels and the respective confidence interval (CI) for each group and specific diagnosis estimated by the KM method. Survival was classified as high if it is greater than or equal to 90%, medium if it is between 70% and 90%, low if it is between 30 and 70%, and very low if it is less than 30%.

The only diagnosis with high survival was in group XIb-thyroid cancer (N=13) with a narrow CI range. The diagnosis with medium survival (greater than 70%) was identified in groups V-retinoblastoma (87.2%), IIa-Hodgkin’s lymphoma (86.8%), Xc-gonadal tumors (83.3%), IId-miscellaneous lymphomas (80%), IVa-nephroblastoma (79.5%), and IIc-Burkitt’s lymphoma (75.4%). The lowest survival was observed in cases with leukemias, in group Ib-acute myeloid leukemia (43.8%), in solid tumor, the group IIId other gliomas (24.3%), and in group IIIf CNS non-specific (28.6%).

### Survival by Age

In **Supplementary Material; Table 3** shows the KM survival rates by groups of age. Differences in survival patterns between age groups, were found in leukemias and in solid tumors of groups III-CNS, VI-renal, X-germ cell, and XI-other tumors. In the case of leukemias, the survival pattern showed an inverted U-shape, where children under 1 year of age and over 15 years old had the lowest survival outcomes. In contrast, for solid tumors, survival by age was U-shaped, with children under one year of age having the best survival. However, this pattern is not consistent across diagnostic groups. In CNS tumors, the survival pattern was not clear, because by subtypes, the number of cases did not allow to define a clear pattern. The same occurred in type X-germ cell tumors and XI-other epithelial tumors. However, for kidney tumors, survival was better for children under 10 years of age.

### Survival by Sex

Survival comparison stratified by sex is shown in **Supplementary Material; Table 4**. Specifically, girls exhibited a lower survival for the following tumors: V-retinoblastoma (15 points lower), Xa-germ intracranial and intraspinal tumors (28 points lower), VIIIc-Ewing’s tumor (20 points lower), and IIIa-ependymomas (almost 20 points lower).

### Survival by Time From Diagnosis

**Supplementary Material, Table 5** shows the survival results according to the time elapsed between perception of symptoms and the formal diagnosis of cancer. No relevant differences were observed in leukemias, while in solid tumors, an inverse relationship was generally observed between the time of diagnosis and survival.

**Figure 2:**
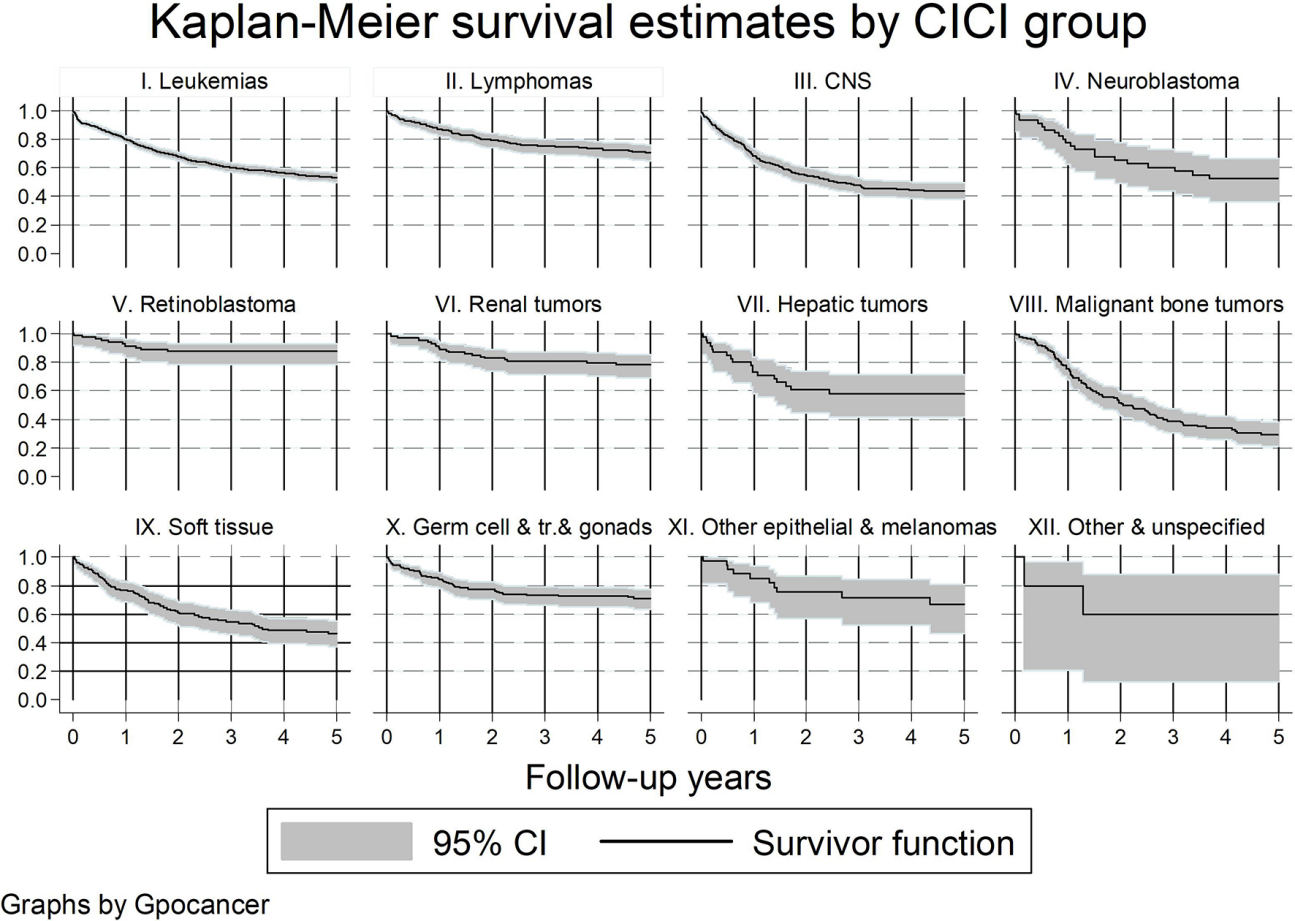
Kaplan–Meier survival curves by cancer diagnosis group.

**Figure 2** shows the KM survival curves by cancer diagnosis.

## Discussion

The 5-year observed survival was 56.1% with a 95% CI [23.9%, 58.1%], and 54.3% 5-year survival weighted by age according to the WHO population.

Regarding Mexico, a previous study as published by Pérez-Cuevas et al. (2013) (14) for a similar pediatric cohort from 2006 to 2009 from the non-IMSS-affiliated population reported an overall 3-year survival of 68.1%, with important regional variations. Differences in 3-year survival were observed between the IMSS-affiliated and non-affiliated populations. In the case of Ia-lymphoid tumors, survival in the IMSS population was higher for non-IMSS affiliated at the national level.

However, these results show a significant gap in relation to the minimum goal proposed by the WHO objective of 60% (4), neither the level achievable by other countries with a similar income level.

Comparing other countries, a study from the periods closest to 2006–2012, we compared with those for United States, published by the National Academies of Sciences, Engineering, and Medicine (6) with data from the SEER (2000–2009); the results are systematically superior when compared to Mexico. The overall cancer survival rates for the IMSS population were 30 percentage points lower than those in the United States.

Regarding other countries with data from population-based cancer registries, we compared our results with a study from Shanghai, China (the biggest city, representing 10% of the population) for the period 2002–2005 (25). In that period, they also reported lower results compared with those in the United States and Europe, with a 5-year observed survival for all childhood cancers combined 55.7% (95% CI: 51.7–59.6%), very similar to our results (56.1%). The 5-year observed survival rate similar for leukemia was (52.2%—Shanghai vs. 54.5%—IMSS), CNS tumors (41.2%—Shanghai vs. 42.7%—IMSS). However, in our study, IMSS had a higher survival rate for lymphoma (58.8% from Shanghai vs. 70.4% from IMSS) and retinoblastoma (75.0% vs. 87.2% IMSS). However, there was a lower survival rate for epithelial cancer (88.9% vs. 69.8% IMSS), malignant renal tumors (86.7% vs. 79.5% IMSS), germ cell and other gonadal tumors (78.4% vs. 72.6% IMSS), soft tissue sarcoma (54.1% vs. 46.6% IMSS), and bone tumors (52.6% vs. 31.9% IMSS).

The cancer diagnoses that require urgent intervention at the IMSS are those showing a gap in the survival rate, which are the cancer groups of leukemias (lymphoid and myeloid), IIb-non-Hodgkin’s lymphoma, III-CNS tumors, VI- kidney tumors, VIII-bone tumors, and XI-soft tissue tumors.

### Results About Survival Patterns

Survival patterns observed according to age was as expected according to the prognostic definition of risk and findings from studies in other countries. Related to survival patterns by sex, similar to previous studies, for the more common cancers, no sex-based pattern was observed. However, a differentiated pattern by sex was reported by Johnston et al. (2010) (26) in the United Kingdom, and for the 1991–1996 cohorts in Williams et al. (2019) (27), based on data from the US SEER.

With regard to V-retinoblastoma, our cohort survival was 93% in male vs. 78% in female patients, while in the UK study by Johnston et al. (2010) (26), no differences were found by sex.

In the case of VIIIc Ewing tumors, coincidentally, Johnston et al.’s study (2010) (26) reported that bone tumors had a worse prognosis among girls, while for neuroblastoma, the same was true for men. In the case of type Xa-tumors intracranial and intraspinal tumors, Johnston et al. (26) reported between 5 and 9 percentage points lower for girls.

In accordance with our results, previous studies in leukemia in children did not find any association pattern in relation to the diagnosis time (28). In the case of lymphomas, contrary to our results, previous studies in the adult population by Zurko et al. (2019) (29)and Nikonova et al. (2015) (30) did not find any relationship between survival and a longer time to diagnosis. Furthermore, in the case of CNS tumors, previous studies reported a shorter time to diagnosis in high-grade tumors than in low-grade tumors (31). In Mexico, Barragán et al. (2020) (32) reported a shorter time to diagnosis (90 vs. 120 days), higher-grade tumor, and lower survival, while Fukuoka et al. (2014) (33) reported the same conclusion in their study in the Japanese population. Thus, these results were consistent with the theory proposed by Porta et al. (1991) (34) on the impact of the biological behavior of the tumor on survival.

### Strengths and Limitations of the Study

The present study reports the highest number of cases from an incidence registry, in the IMSS of Mexico. Among the limitations that we must mention are the loss to follow-up of 18.4%, which exceeds 25% for patients >15 years of age and patients residing in provincial areas. The loss of follow-up by age is associated with the loss of national healthcare eligibility. This is because in Mexico, the law indicates that, upon reaching 18 years of age, patients are required to demonstrate that they are financially dependent due to disability or because the minor is an active student, and the latter among cancer patients is difficult to achieve. Therefore, it can be expected that the results are overestimated for the diagnoses of poor prognosis among children over age 15 and vice versa.

We have two estimation issues related to the socioeconomic status of families. On the one hand, the underreporting of incident cases includes those who die before confirming the diagnosis; we think that it must be the cases that had problems accessing medical care, which is expected in marginalized areas of the country. The cancer registry includes population from those areas (Chiapas, Guerrero, and Hidalgo mainly). On the other hand, for those children diagnosed and treated in private health services, in Mexico, it is only possible for very wealthy families in the country. That is, we have estimation problems from the two extremes of the socioeconomic population distribution.

In a previous study on social inequalities in the survival of children with leukemia (35), we have more detailed socioeconomic data of the families of the children included in the RCC of the IMSS. When comparing the income distribution of the families included in the registry against the income distribution of the entire IMSS-insured population in the central region, it was observed that the median income was similar, but the dispersion was different. People with a monthly income less than USD 105 was 0.5% in the total IMSS population vs. 8.8% in the cancer registry population; for incomes greater than USD 750, it was 23.6% in the total IMSS population vs. 8.5% in the cancer registry population; and for incomes over 1,500, it was 7.5% for the IMSS population vs. less than 1% on the cancer registry population. Thus, we think the population included in the cancer registry is underrepresented for families with the highest incomes and overrepresented for families with the lowest incomes.

Regarding the potential bias that this may have on the survival results, in our results, we observed that all deaths from families in the fourth income quartile occurred in the tertiary hospitals of the IMSS, while this proportion was only 73% for families in first quartile. From this, we suppose that attrition in the fourth quartile may have been due to survivors. Therefore, survival could be underestimated for children from the richest families (6% of cases: 1%-7.5%), while survival may be overestimated for the most disadvantaged population (16.1%: 8.5%-23.6%). Because of this crossover effect, we are hesitant to say the direction of the effect on survival estimates.

### Future Studies

Further multivariate analysis is necessary to analyze interactions or modulatory effects among a patient´s characteristics, including the behavior and grade of tumors, which will allow us to better understand this relationship. To provide continuity to these initial results, it is necessary to study the differences regarding clinical management; other studies have pointed out the importance of studying the mortality associated with treatment, its associated toxicity, the availability of procedures such as bone marrow transplantation, appropriate indication and adherence to treatment, and the opportunity for immunogenetic studies (6,8). It should also be noted that no quality information was available on the causes of death; thus, the calculation of net survival could not be made. Future studies should be carried out to systematically evaluate this information and improve its quality. Finally, an in-depth study of the effect of socioeconomic variables, access to services, and specifically, the impact of the delay of diagnosis on survival is necessary.

## Conclusion

Survival results in the 2006–2012 Mexican cohorts show a significant gap in relation to the maximum level achievable and still below the goal proposed by the WHO for 2030.

## Data Availability Statement

The original contributions presented in the study are included in the article/**Supplementary Material**. Further inquiries can be directed to the corresponding authors.

## Ethics Statement

The studies involving human participants were reviewed and approved by [Comité Local de Investigación del Hospital de Pediatría CMN SXXI] Research and Bioethics Committee of the Pediatric Hospital, CMN SXXI IMSS (registration number 2003/718/070). Verbal informed consent was required. Written informed consent from the participants’ legal guardian/next of kin was not required to participate in this study in accordance with the national legislation and the institutional requirements.

## Author Contributions

AC designed the study, analyzed the data, and prepared the draft of the manuscript. SM collaborated in the critical analysis of the methodology and results and in the preparation of the manuscript. All authors read and approved the final manuscript.

## Funding

No special financing was obtained for carrying out this study. Financing from Hospital Infantil de México Federico Gómez was provided to cover publication costs.

## Conflict of Interest

The authors declare that the research was conducted in the absence of any commercial or financial relationships that could be construed as a potential conflict of interest.

## Publisher’s Note

All claims expressed in this article are solely those of the authors and do not necessarily represent those of their affiliated organizations, or those of the publisher, the editors and the reviewers. Any product that may be evaluated in this article, or claim that may be made by its manufacturer, is not guaranteed or endorsed by the publisher.
