# Supplementary material for: Childhood Cancer Survival, 2006-2012 Cohorts of Mexican Institute of Social Security Beneficiaries at the Central-South Region of Mexico
Source: Front Oncol. 2022 Jul 1;12:882501. doi: 10.3389/fonc.2022.882501 (PMC9283836; doi:10.3389/fonc.2022.882501)
Supplement: Supplementary file 1 [file DataSheet_1.docx]

1. **Kaplan–Meier survival curves by age-group, general and for CICI groups with differences statistically significant**

Log rank test for equality of survivor functions: p < 0.05

1. **Kaplan–Meier survival curves by time from diagnosis, general and for CICI groups with differences statistically significant**

Log rank test for equality of survivor functions: p < 0.05
